# Stimuli-responsive platinum and ruthenium complexes for lung cancer therapy

**DOI:** 10.3389/fphar.2022.1035217

**Published:** 2022-10-17

**Authors:** Cheng Zhang, Tong Kang, Xinyi Wang, Jiaqi Song, Jia Zhang, Guanying Li

**Affiliations:** ^1^ The Department of Thoracic Surgery, The First Affiliated Hospital of Xi’an Jiaotong University, Xi’an, Shaanxi, China; ^2^ Department of Dermatology, The Second Affiliated Hospital of Xi’an Jiaotong University, Xi’an, Shaanxi, China; ^3^ Department of Biophysics, School of Basic Medical Sciences, Xi’an Jiaotong University, Xi’an, Shaanxi, China

**Keywords:** stimuli-responsive, metallic prodrugs, lung cancer, Pt anticancer drugs, Ru anticancer drugs

## Abstract

Lung cancer is the most common cause of cancer-related deaths worldwide. More efficient treatments are desperately needed. For decades, the success of platinum-based anticancer drugs has promoted the exploration of metal-based agents. Four ruthenium-based complexes have also entered clinical trials as candidates of anticancer metallodrugs. However, systemic toxicity, severe side effects and drug-resistance impeded their applications and efficacy. Stimuli-responsiveness of Pt- and Ru-based complexes provide a great chance to weaken the side effects and strengthen the clinical efficacy in drug design. This review provides an overview on the stimuli-responsive Pt- and Ru-based metallic anticancer drugs for lung cancer. They are categorized as endo-stimuli-responsive, exo-stimuli-responsive, and dual-stimuli-responsive prodrugs based on the nature of stimuli. We describe various representative examples of structure, response mechanism, and potential medical applications in lung cancer. In the end, we discuss the future opportunities and challenges in this field.

## 1 Introduction

Lung cancer (LC) is the most common malignancy and the top leading cause of cancer-related death worldwide (Govindan et al., 2006; Sung et al., 2021). Due to the lack of early clinical symptoms, most patients with LC are diagnosed at an advanced stage, resulting in the loss of opportunity for surgical treatment (Wang et al., 2015a). Platinum(II)-based chemotherapy is one of the pillars of clinical treatment for advanced LC (Chang, 2011; Pirker, 2020). The success of platinum-based anticancer drugs sparks interest in other metal-based anticancer drugs. Despite the dominance of small organic molecules and bio-derived compounds in the pharmaceutical market, metallic drugs continue to attract attention for their unique properties. In addition, four ruthenium complexes by far have successfully entered clinical trials and exhibited inspiring therapeutic effect on lung cancer metastasis.

Despite the great success of cisplatin in clinical usages, it still has some drawbacks, including significant side effects due to systemic toxicity, primary and acquired resistance, and low bioavailability for various reasons (Lippard, 1982; Wang and Lippard, 2005; Wang, 2010; Wang and Guo, 2013). Therefore, the development of metallic drugs urgently requires new strategies that can address these issues in a targeted manner. Prodrugs may be a potential strategy that can address the current metallic drugs dilemma. Prodrugs are inactive derivatives of a drug that are biotransformed *in vivo* to produce an active form for targeted drug delivery and therapy (Rautio et al., 2008; Bildstein et al., 2011; Redasani and Bari, 2015). To date, prodrugs have been at the forefront of improving the targeting and bioavailability of drugs and improving their physiochemical, pharmaceutical, and pharmacokinetic properties (Kratz et al., 2008; Mazzaferro et al., 2013; Walther et al., 2017).

Analog to metal complexes-based anticancer drugs, Pt- and Ru-based prodrugs hold advantages in diversity that is not usually available with organic molecules. First, they maintain a three-dimensional structure with square-planar or octahedral conformation (Hambley, 2007a). The different geometries confer different biological activities to the complexes. Second, bioactive ligands can be easily linked with the metal ions *via* coordination or modification of chelated ligands, and exhibit new pharmacological properties (Bruijnincx and Sadler, 2008). Third, Pt- or Ru-based complexes that undergo ligand exchange offer unique action for killing cancer cells. As a presentative example, cisplatin undergoes activation through hydrolysis inside cells to bind to DNA, suppressing DNA replication (Ghosh, 2019). Ligand exchange reaction of metal complexes bearing bioactive molecules as coordinated ligands is also exploited for delivery purpose. In addition, rich photochemical and photophysical properties of Pt- or Ru-based complexes are beneficial in designing “smart” prodrugs with stimuli-responsive characteristics, and expanding their utility beyond therapeutic agents (Heffern et al., 2014; Ma et al., 2014). Therefore, the diversity and variability of molecular structures provide a broad platform for the design of responsive prodrugs based on Pt- and Ru-complexes.

One of the most important aspects of prodrug activation is the appropriate trigger (Zhang et al., 2017; Gu et al., 2018), which can be divided into two main categories: endogenous and exogenous stimuli (Chen et al., 2016a; Liu et al., 2016; Yang et al., 2016; Brun et al., 2017; Hu et al., 2017). Endogenous stimuli include redox gradients, pH values, enzyme concentrations, hormone levels, glucose concentrations, etc., usually related to pathological characteristics. Exogenous stimuli, such as light, temperature, magnetic fields, high-energy radiation, and ultrasound, remotely control prodrug activation by means outside the organism. In addition to the single triggers mentioned above, different triggers can act synergistically with each other to accomplish the activation of the drug. Therefore, the structural characteristics of the parent drug and its function in response to stimuli are prerequisites for the action of the prodrug. For metal drugs, the versatility of molecular structure and function makes them more likely to be qualified stimulus-responsive prodrugs (Van Rijt and Sadler, 2009; Schatzschneider, 2010; Graf and Lippard, 2012). Some recent studies have also shown the potential of some stimulus-responsive metal drugs to enhance pharmacological effects and reduce side effects, for example, some inactive complexes of metal ions in the oxidized state can be reductively activated in the reducing environment of the pathological state and transformed into the active form to exert their pharmacological effects (Van Rijt and Sadler, 2009; Romero-Canelon and Sadler, 2013).

Due to the great success of Pt- and Ru-based metallodrugs in clinical usages or in clinic trials, in this review we aim to summarize and analyze the representative stimulant-responsive Pt- and Ru-based agents in lung cancer therapy. These metallic anticancer agents are classified into three categories: endo-stimuli-responsive drugs, exo-stimuli-responsive drugs, and multi-stimuli-responsive drugs. The different stimulatory modalities are described in terms of design strategies, molecular structures, mechanisms of action and corresponding clinical applications. Finally, we discuss the future opportunities and challenges in this field.

## 2 Endo-stimuli-responsive Pt- and Ru-based anticancer drugs

Tumor development is a dynamic process involving a continuous interaction between tumor cells and the tumor microenvironment (Jin and Jin, 2020). Compared with normal tissues, tumor tissues have unique pathophysiological hallmarks, such as hypoxia, reducing environment, low pH, elevated reactive oxygen species (ROS) levels, elevated intracellular glutathione (GSH) levels, and abnormal expression of specific enzymes (Jin and Jin, 2020). These unique factors can act as endogenous triggers of the prodrug, specifically triggering the conversion of the prodrug and exerting its pharmacological effects (Mura et al., 2013; Gulzar et al., 2015). A large number of endo-stimuli-responsive metallic drugs triggered by redox, pH, and specific enzymes have been developed for these abnormal factors (Van Rijt and Sadler, 2009; Schatzschneider, 2010; Scaffidi-Domianello et al., 2011; Graf and Lippard, 2012; Ding et al., 2014). In this section, these metallic drugs are outlined according to different microenvironmental factors.

### 2.1 Redox-responsive

The difference in redox potential between tumor tissue and normal tissue is widely recognized, and this difference can serve as a trigger for redox-responsive prodrugs (Schafer and Buettner, 2001; Brun et al., 2017). For example, glutathione (GSH) is an important intracellular reducing agent. GSH/GSH disulfide (GSSG), the major redox pair in mammalian cells, is mainly involved in the maintenance of thiol-containing enzyme stability, maintenance of cell membrane integrity and avoidance of ROS-induced damage. The intracellular GSH concentration is about 2–10 mM, while the concentration of GSH in body fluids is lower (2–20 μM) (Hong et al., 2006; Cheng et al., 2011a). The high intracellular GSH concentration maintains a highly reductive intracellular environment. Moreover, the rapid proliferation of tumor cells resulted in a more than four times higher GSH content in tumor tissues than in normal tissues, creating a stronger reductive environment (Saito et al., 2003). The strongly reducing environment can trigger redox-sensitive prodrugs to release their active forms inside tumor cells to exert their pharmacological effects. In addition to GSH, some other bioreductants such as ascorbate can also act as triggers to activate redox-responsive drugs.

Metal ions such as Pt, Ru, Co and Fe have multiple oxidation states that can be regulated by ligands so that they can be activated in the reducing environment in the pathological state and thus release the active form of the drug. Thus, the redox reactivity of metal complexes has become one of the most successful classes of stimuli-responsive metal drugs designed.

#### 2.1.1 Platinum(IV) prodrugs

**FIGURE 1:**
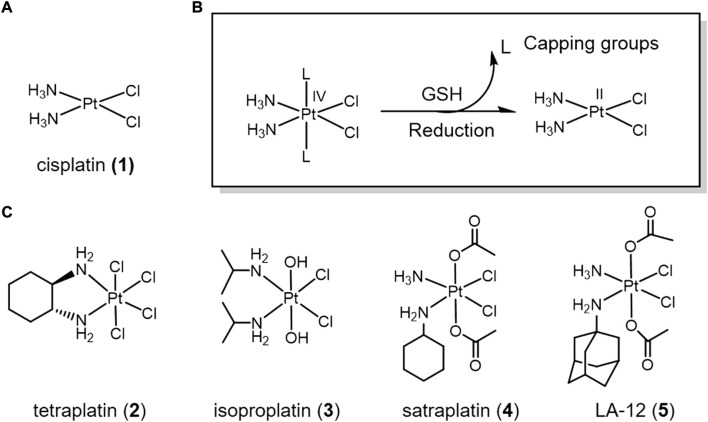
**(A)** Chemical structure of *cis*-platin. **(B)** Action mechanism of redox-responsive Pt(IV) prodrugs undergoing GSH reducible activation to form active Pt(II) drugs. **(C)** Pt(IV) prodrugs that have entered clinical trials.

Cisplatin (**1**) (Figure 1), the most successful metallic anti-cancer drug, is routinely used worldwide for the treatment of many types of cancer (Mjos and Orvig, 2014). However, classical Pt(II) drugs also suffer from severe side effects, drug resistance and systemic toxicity (Hambley, 2007b; Klein and Hambley, 2009). Pt(IV) complexes are believed to overcome the defects of classical Pt(II) drugs as prodrugs (Bruijnincx and Sadler, 2008). The hexa-ligand octahedral conformation of Pt(IV) is kinetically inert, which reduces off-target effects prior to binding to DNA and thus reduces systemic toxicity (Hall and Hambley, 2002). Pt(IV) prodrugs can be reduced to the Pt(II) form by the elimination of two axial ligands by the reducing agent, which is activated at the tumor site (Hall et al., 2007; Venkatesh and Sadler, 2018). Therefore, the redox potential at the tumor site is an important factor affecting the efficiency of Pt(IV) complex activation. In addition, the alteration of the two axial ligands can regulate the reduction potential and cytotoxicity of Pt(IV) complexes, and also provide targeting functions (Ding and Bierbach, 2015; Basu et al., 2016). Based on this, Pt(IV) complexes can be classified into three categories according to the nature of the ligands: 1) axial ligands lacking biological activity, such as chloride, hydroxide, etc.; 2) axial ligands with biological activity, such as enzyme inhibitors, etc.; 3) axial positions connected to other targeting carriers.

A series of positive results have been achieved in the development of Pt(IV) complexes lacking bioactive ligands. Many Pt(IV) complexes have been reported as potential anticancer drugs. Four of these complexes even entered clinical trials, namely complexes tetraplatin (**2**), isoproplatin (**3**), satraplatin (**4**), and LA-12 (**5**) (Figure 1) (Wexselblatt and Gibson, 2012). Tetraplatin (**2**) was the first Pt(IV) drug to enter clinical trials (Schilder et al., 1994). However, due to severe neurotoxicity, tetraplatin was discontinued in phase I clinical trials (Tutsch et al., 1999). Because of high stability of the hydroxide axial ligands, isoproplatin (**3**) is relatively difficult to be reduced by bioreductants. Phase III clinical trials showed that isoproplatin did not exhibit greater anticancer activity than cisplatin or carboplatin (Anderson et al., 1988). Satraplatin (**4**) was the first reported oral platinum-based anticancer agent and has been tested in multiple clinical trials (Bhargava and Vaishampayan, 2009). LA-12 (**5**) has completed a Phase I clinical trial with positive results (Zak et al., 2004). Unfortunately, these Pt(IV) prodrugs have been abandoned for clinical approval due to low efficacy and severe side-effects.

**FIGURE 2:**
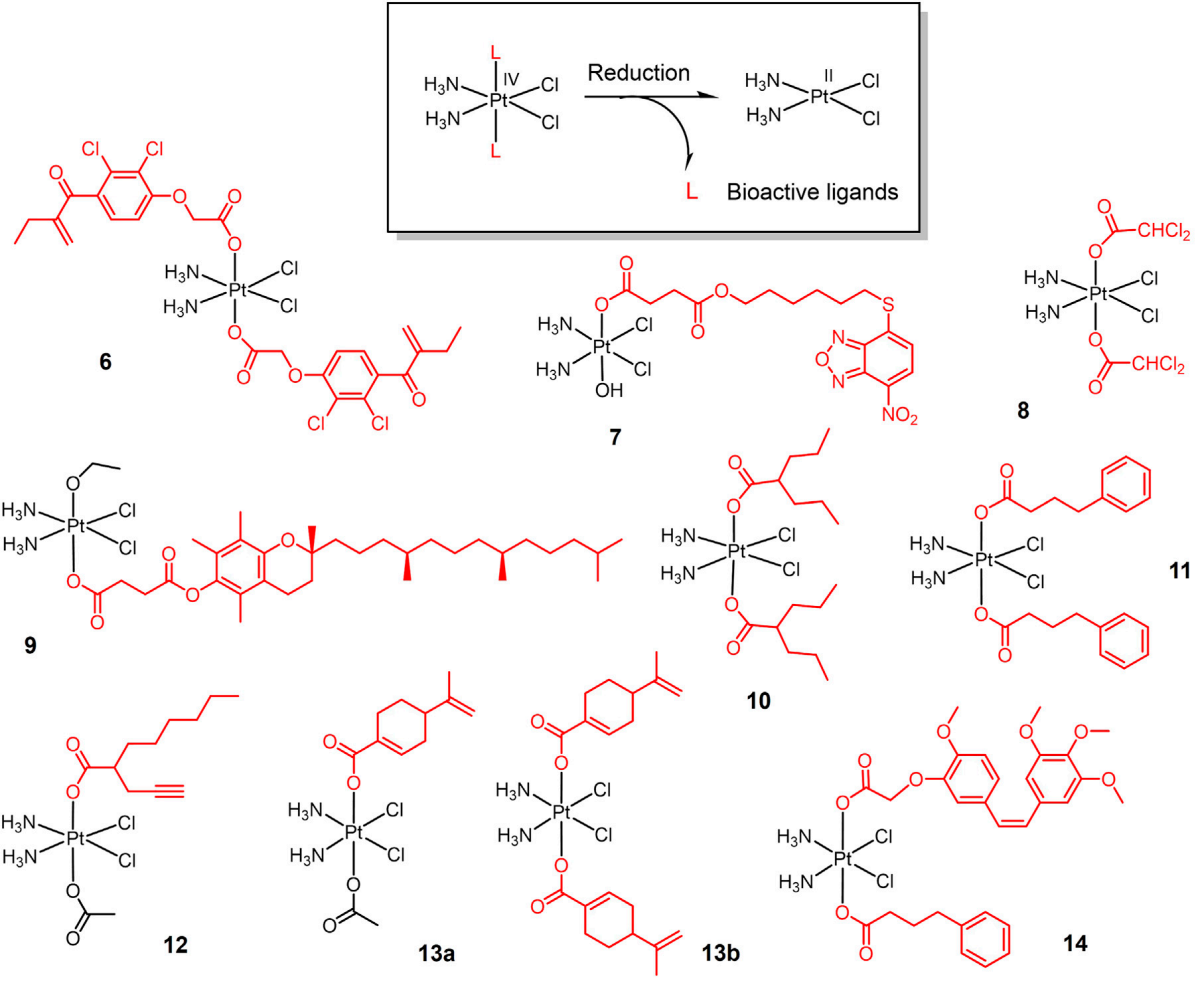
Redox-responsive Pt(IV) prodrugs undergo reduction to form active Pt(II) drugs accompanied by the release bioactive molecules. Complexes **6** and **7** bear glutathione-S-transferase inhibitors; Complex **8** bears dichloroacetate as pyruvate dehydrogenase kinase inhibitor; complex **9** bears α-TOS as inhibitor for Bcl-xL-Bax protein-protein interaction; complexes **10-12** bear histone deacetylase inhibitors (HDACi); Complexes 13a,b bear perillic acid as anti-metastatic molecule; and complex 14 bears dual bioactive ligands: phenylbutyrate as HDACi and CA4 as tubulin polymerization inhibitor, respectively.

Bioactive ligands are introduced to Pt(IV) prodrugs for seeking high anticancer efficacy in lung cancer (Figure 2). Overexpression of glutathione-S-transferase (GST) leads to cisplatin resistance (Nakajima et al., 2003). Etanercept (EA), a broad-spectrum GST inhibitor, can reverse GST-mediated cisplatin resistance (Takamatsu and Inaba, 1992). An EA-Pt (IV) complex **6** was developed based on this (Ang et al., 2005). Complex **6** inhibited GST activity in lung cancer A549 cells more strongly than EA and cisplatin alone. Complex **6** exhibited inhibitory effects on cisplatin-resistant A549 cells. Given that the long-term efficacy of EA is limited by toxicity due to diuresis and fluid imbalance (Ploemen et al., 1994), and that EA is readily transported out of the cell by specific pumps (Townsend and Tew, 2003), NBDHEX, a new GST inhibitor with stronger GST inhibition than EA and not transported by transport pumps (Ricci et al., 2005; Federici et al., 2009), was conjugated to oxoplatin *via* a succinic anhydride reaction to yield a new Pt(IV) complex **7** (Chen et al., 2019). Complex **7** showed stronger tumor suppression efficacy and less biotoxicity than cisplatin in animal studies.

Based on the same strategy, a new Pt(IV) complex, named mitaplatin (**8**) with two dichloroacetate (DCA) axial ligands was developed (Dhar and Lippard, 2009). DCA is an inhibitor of pyruvate dehydrogenase kinase (Stacpoole et al., 2003). DCA promotes the release of cytochrome c (cyt c) and nuclear translocation of apoptosis-inducing factor (AIF) by altering the mitochondrial metabolism of tumor cells, ultimately leading to apoptosis without affecting normal cells (Bonnet et al., 2007). Thus, Complex **8** shows a dual-killing mode which targets the nuclear DNA with released cisplatin and mitochondria with released DCA. Complex **8** exhibited comparable cytotoxicity to cisplatin in lung cancer cells and did not induce normal cell death at the same concentration.

Inspired by the dual killing mode of complex **8**, a new dual-targeted Pt(IV) complex **9** was developed using the vitamin E analogue α-tocopheryl succinate (α-TOS) as an axial ligand (Suntharalingam et al., 2014). α-TOS disrupts Bcl-xL-Bax interactions, thereby inducing mitochondria-mediated apoptosis (Neuzil, 2003; Shiau et al., 2006). Complex **9** exhibited more potent killing ability than cisplatin against lung cancer cells. And the cellular uptake capacity of this complex was 15–20 times higher than that of cisplatin.

The combination of histone deacetylase inhibitors (HDACi) with platinum-based anticancer drugs has received much attention (Bots and Johnstone, 2009; Hrebackova et al., 2010). HDACi increases the acetylation level of histones, hinders the interaction of histones with DNA, and exposes nuclear DNA to DNA-damaging chemotherapeutic drugs (Bolden et al., 2006; Buchwald et al., 2009). The Pt(IV) complex is well suited for this combination therapy, reducing the side effects of traditional Pt(II) drugs while exert the chemosensitizing effect of HDACi. Based on the above idea, various Pt(IV) complexes with HDACi as axial ligand were developed. Valproic acid (VA)-Pt(IV) complexes (**10**) (Figure 2) are the first complexes of this type (Yang et al., 2012). Complex **10** showed enhanced antitumor activity *in vitro* and *in vivo*, and animal experiments confirmed the lower nephrotoxicity of complex **10** *in vivo*.

The initial success of HDACi complexes has prompted researchers to further explore better HADCi complexes. Complex **11** contains two phenylbutyrate (PhB) groups as HADCi and shows nearly 50-fold higher cytotoxicity than cisplatin against A549 cells (Raveendran et al., 2016). Complex **12** contains a racemic mixture of 2-(2-propynyl) octanoic acid (POA) along with an inert acetate as axial ligands, and shows a better anticancer activity *in vivo* on Lewis lung carcinoma (LLC)—bearing mice than cisplatin (Gabano et al., 2017).

Perillic acid (PA), an active metabolite of limonite that showed anti-metastatic effects, was used as a bioactive capping ligand for the Pt(IV) complexes **13a-b** (Ravera et al., 2021). The combination of PA with cisplatin can significantly improve the efficacy of cisplatin. The complexes were stable in buffer solution and were reduced in the cytoplasmic lysate of A549 cells, decomposing cisplatin and PA to exert antitumor effects. The complexes exhibited an order of magnitude stronger cytotoxicity than cisplatin against A549 cells in multicellular spheroids model.

Combretastatin A-4 (CA4), an tubulin polymerization inhibitor that exhibits dramatically 1000-folds more cytotoxic than cisplatin, was conjugated with Pt(IV) (**14**). CA4 showed higher cytotoxicity than Pt(IV)-CA4 conjugates *in vitro*, while *in vivo* test showed that Pt(IV)-CA4 conjugates exhibited significantly enhanced tumor suppression and reduced acute toxicity (weight loss) in LLC-bearing mice (Schmidt et al., 2021). Pt(IV)-bioactive ligand conjugates usually exerted synergistic anticancer activity *via* multi-action. In case of extremely potent bioactive ligands such as CA4, however, Pt(IV) moiety merely served as a self-immolative carrier for CA4, considered as prodrug for CA4 rather than multi-action prodrug.

#### 2.1.2 Ruthenium prodrugs

Ruthenium has a distorted octahedral geometry with II to IV oxidation states (Merlino, 2016; Zeng et al., 2017a). The potential biological activity of ruthenium complexes was first explored in the 1950s (Dwyer et al., 1952), and in the 1980s, Clark et al. reported the potential targeted anticancer activity of Ru(III) complexes (Clarke et al., 1980). Ru(III) complexes can only be activated reductively in tumor tissue under hypoxic and low pH conditions. Several ruthenium-based complexes have shown significant efficacy against lung cancer in recent decades, and some of them have demonstrated lower side effects than cisplatin.

**FIGURE 3:**
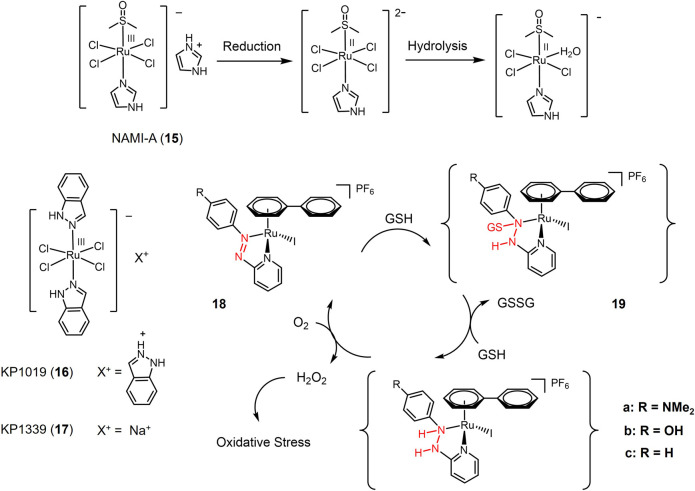
Redox-responsive Ru(III) complexes NAMI-A (**15**), KP1019 (**16**) KP1339 (**17**) that have entered clinical trials, and Ru(II) complexes with redox-active azole-ligand (**18a-c**).

The first ruthenium-based anti-cancer candidate in clinical trials is NAMI-A (**15**, seen in Figure 3) (Cocchietto et al., 2003; Bergamo and Sava, 2011; Leijen et al., 2015). NAMI-A is a reducible Ru(III) complex with a heterocyclic N-donor ligand. NAMI-A has low activity against primary tumors, but has demonstrated high activity against lung metastases *in vivo*. Mechanistic studies revealed that NAMI-A can be reduced by GSH or ascorbic acid under physiological conditions to form the Ru(II) active form, enhancing the anti-metastatic activity of NAMI-A, which is similar to the principle of Pt(IV) prodrug (Levina et al., 2009). Phase I/II clinical study of NAMI-A revealed that NAMI-A showed selective effect on lung metastasis of solid metastasizing tumors (Sava et al., 2003; Leijen et al., 2015). Two NAMI-A analogues, KP1019 (**16**) (Hartinger et al., 2008) and KP1339 (**17**) (Trondl et al., 2014), have also entered clinical trials.

In recent years, a series of Ru(II) complexes have been of interest to researchers as redox-responsive anticancer drugs. In contrast to Ru(III) complexes that are activated through Ru(III) to Ru(II) reduction (Figure 3), the Ru(II) complexes are reducibly activated through catalyzing the redox-active ligands reduction (Zhang and Sadler, 2017). Ru(II) complex **18** contained azopyridine derivatives, whose reduction potentials significantly decreased *via* azopyridine-Ru(II) coordination. Azo group in complex 18 was capable to be reduced by GSH forming GSH additive intermediate (19) and catalyzed a redox reaction between GSH with O_2_ generating high level of intracellular H_2_O_2_ that produced oxidative stress to cause cancer cell death (Dougan et al., 2008). Complex **18a** bearing electron-donating substituent-NMe_2_ exhibited the highest cytotoxicity against A549 cells with a e value of 2 μM.

### 2.2 pH- responsive

The pH gradient between biological systems reflects the different acid-base environments of the body (Felber et al., 2012; Crucho, 2015). The extracellular pH of tumor tissues is usually 5.6–6.8, which is significantly lower than that of normal tissues (pH 7.4) due to altered and accelerated metabolic patterns (Gatenby and Gillies, 2004). Based on this, pH becomes a possible endogenous trigger for stimulation of reactive prodrugs. Among all pH-responsive metallodrugs, two main strategies have been adopted to design response systems: pH-labile chemical bonds and pH-sensitive nanocarriers.

**FIGURE 4:**
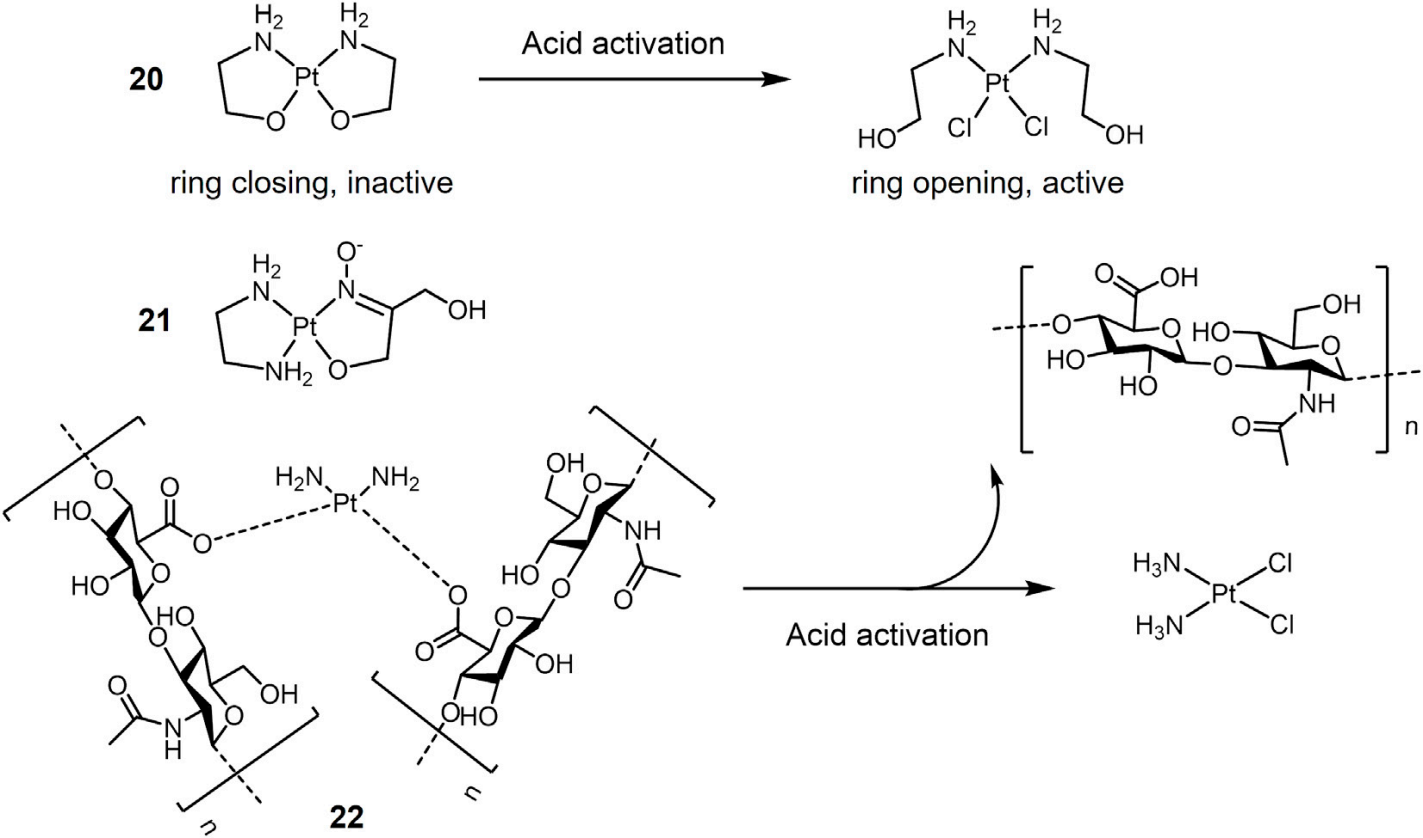
pH-responsive Pt(II) complexes undergoing acid activation.

A representative class of pH-sensitive platinum complexes in lung cancer therapy is the Pt(II) complex containing two chelated amino alcohol ligands (Galanski et al., 2003; Galanski et al., 2004; Scaffidi-Domianello et al., 2011). Platinum complexes with N, O chelating ligands are inert at physiological pH, but can be activated by a microacidic environment and converted to highly cytotoxic platinum complexes. As shown in Figure 4, Pt(II) complex **20** chelated with two aminoethanolate forming closed-ring at physiological pH, and exhibited low toxic effect against A549 cells with an IC_50_ value of 115 μM under pH 7.4. Acidic condition triggered the ring opening of complex **20**, resulting in the active Pt(II) species. Under pH 6.0, complex **20** showed significant cytotoxicity against A549 cells with an IC_50_ value of 41.2 μM (Galanski et al., 2004). Complex **21** with a 1,3-dihydroxyacetone oxime ligand (Scaffidi-Domianello et al., 2011) performed a similar acid-activation mechanism and showed enhanced cytotoxicity against cisplatin-sensitive A549 cells in an acidic environment (pH 6.0) mimicking solid tumor.

Encapsulation of metallic drugs into pH-responsive drug delivery vehicles is another design strategy for pH-responsive drugs (Taghizadeh et al., 2015; Zhang et al., 2016; Hu et al., 2017). One possible approach is based on the strategy of oxygen donor exchange of chloride ions with carboxylate in cisplatin (Oberoi et al., 2011; Xia et al., 2011; Huang et al., 2012; Fan et al., 2015; Fu et al., 2015; Saisyo et al., 2016). This system is stable at physiological pH, and at acidic condition Pt-carboxylate bonds break, which in turn releases the active platinum drug. Based on this strategy, polyhyaluronic acid (HA) is used as a carrier for cisplatin (Figure 4). Pt(II) forms complex **22** by internal cross-linking with the carboxylic acid of HA through ionic interactions (Fan et al., 2015). The complex exhibit pH-responsive release of cisplatin. The release of cisplatin is 2–3 times higher in acidic pH than in neutral pH. In addition, HA is the major extracellular matrix of tumor tissue and its receptor CD44 is highly expressed in many tumor tissues (Nikitovic et al., 2015). The complex is internalized by CD44-based endocytosis upon arrival in the tumor tissue, and the internalized complex rapidly releases cisplatin in the acidic lysosomal compartment.

pH-sensitive calcium carbonate nanoparticles (CPNP) have also been used as delivery vehicles for cisplatin (Bastakoti et al., 2013; Chen et al., 2013; Chen et al., 2014; Ding et al., 2017). Biomineralization of cisplatin using CPNP produces pH-responsive nanoparticles that are stable at physiological pH and degrade at acidic pH, thereby releasing cisplatin (Chen et al., 2013; Chen et al., 2014). Biomineralized cisplatin particles exhibit significant antitumor activity in A549 tumor-bearing mice.

### 2.3 Enzyme- responsive

The key role of enzymes in biological and metabolic processes is well recognized. In pathological conditions such as tumors, the concentration and activity of certain enzymes are significantly higher than in normal conditions due to changes in enzyme expression (Zelzer et al., 2013; Che and Van Hest, 2016; Karimi et al., 2016). Such abnormalities in enzyme expression and activity can be used as triggers for metallodrug enzyme response systems. Enzyme-responsive drugs usually depend on the cleavage of specific modifications on the drug by the abnormal enzyme.

**FIGURE 5:**
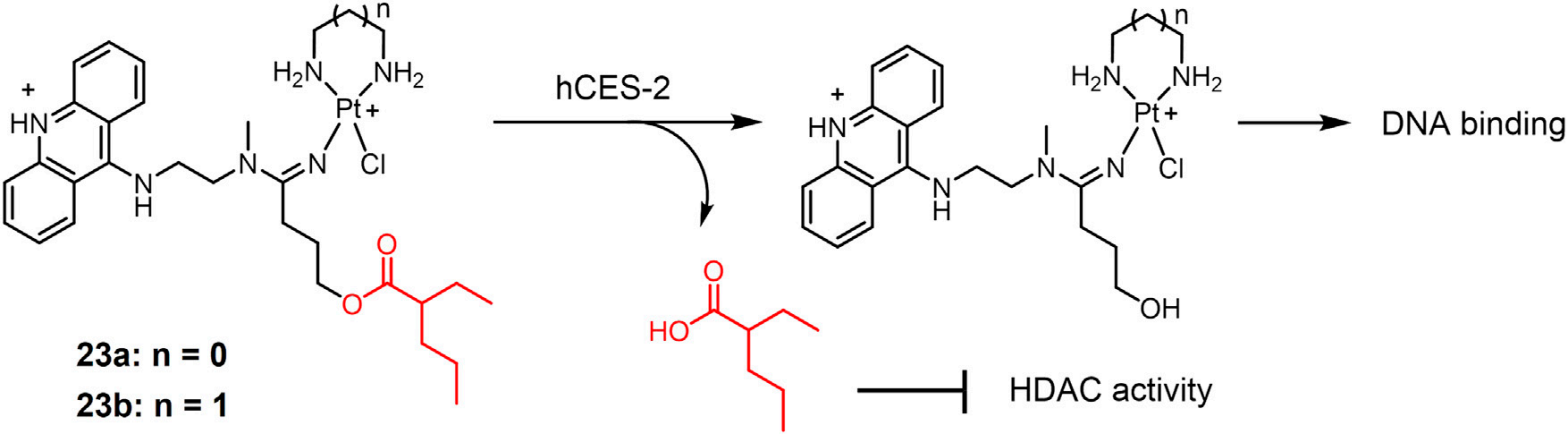
Human carboxylesterase-2 (hCES-2)-responsive Pt(II) prodrugs undergoing enzymatic cleavage to release active Pt(II) drugs and valproate as an HDACi.

A new class of platinum-acridine anticancer drugs has recently been reported as enzyme-responsive prodrugs (Ding et al., 2014)12231. The platinum-acridine complexes **23** (Figure 5) used ethane-1,2-diamine (**23a**) or propane-1,3-diamine (**23b**) as masked groups of Pt(II) complex, respectively (Ding et al., 2014). Valproic ester is recognized as a reactive substrate by human carboxylesterase-2 (hCES-2), which is highly expressed in tumor tissues (Huttunen et al., 2011). Complexes **23a** and **23b** showed complete resistance to chemical hydrolysis but could be efficiently cleaved by hCES-2. Furthermore, the cytotoxicity of complexes **23a** and **23b** was significantly lower than that of the unmodified platinum-acridine complex, which may be due to the reduced reactivity of the modified complex with cellular DNA. The nature of the non-masked groups hardly affects their activity. *In vitro* experiments showed that the activity of both complexes was significantly enhanced in the NCI-H1435 cell line compared to that in the A549 cell line, due to the fact that NCI-H1435 cells possess significantly higher levels of hCES-2 enzyme than A549 cells and that higher levels of hCES-2 perform stronger cleavage of valproate to produce the active form of platinum-acridine hybrids. These findings provided a new idea for the enzymatic conversion of inactive platinum prodrugs to the active form.

## 3 Exo-stimuli-responsive Pt- and Ru-based anticancer drugs

In contrast to the endogenous stimuli of pathological factors in the tumor microenvironment described above, exogenous stimuli such as light, temperature and magnetic fields can activate drugs remotely or *in vitro* at specific times. Therefore, the use of exogenous stimuli to achieve activation of prodrugs can effectively reduce systemic toxicity and side effects. In this section, we will focus on anti-lung cancer metallic drugs activated by light or heat.

### 3.1 Light- responsive

Light as a trigger for stimulating reactive drugs has high temporal and spatial accuracy and is non-invasive (Butler and Sadler, 2013; Mari et al., 2015). And the release profile of the active drug can also be tuned by adjusting the wavelength and intensity of light and the irradiation time. At this stage, there are two main strategies to design photo-responsive metal drugs: 1) photoactivated chemotherapy (PACT) that a prodrug is activated by light to produce bioactive forms; 2) photodynamic therapy (PDT) that a photosensitizer (PS) is irradiated to produce ROSs to induce cell death. PDT is currently being used clinically in oncology treatment (Dolmans et al., 2003). However, PDT is only effective in the presence of oxygen, which limits the use of PDT because of the hypoxia of tumor cells. For both light activation actions, NIR light is always more suitable for *in vivo* use than UV and visible light because of its excellent tissue penetration (Rai et al., 2010; Staderini et al., 2015).

#### 3.1.1 Photo-active platinum complexes

**FIGURE 6:**
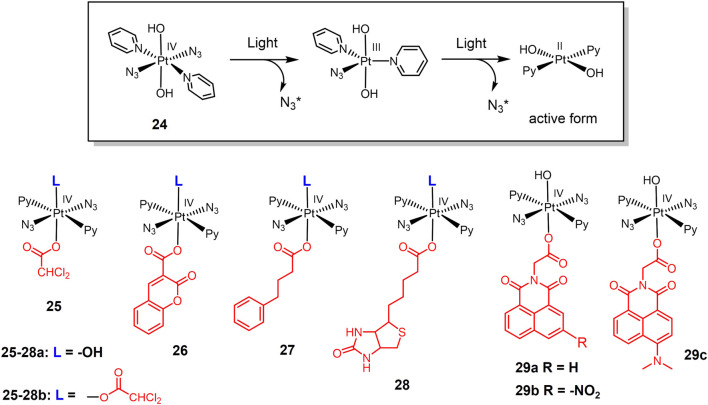
Light-responsive Pt(IV) prodrugs as PACT agents.

The development of photoactive Pt(IV) prodrugs has been reported by researchers since 1996 (Kratochwil et al., 1996). A Pt(IV) complex trans,cis-[PtCl_2_I_2_(en)] was photolyzing through 410 nm light irradiation to form active cis-[PtCl_2_ (en)] anticancer agent that bound to DNA and inhibited tumor cell growth *in vitro*. However, even in the absence of light, this complex was prone to decomposition. To solve this problem, Pt(IV)-azide complexes have entered the vision of researchers. Azide groups substituting iodine ligands in Pt(IV) complexes can improve the stability of these complexes to reducing agents (Butler and Sadler, 2013). Interestingly, all trans Pt(IV)-azide complexes photoactive prodrugs were found to exhibit higher water solubility as well as stronger photocytotoxicity (Mackay et al., 2006). Based on this, a new Pt(IV)-azide complex **24** (Figure 6) was developed (Farrer et al., 2010). Complex **24** was potent cytotoxic to tumor cells under irradiation of low doses of visible light. Investigation of photohydrolytic products had revealed azido ligands were released to bind DNA, while the pyridine ligands remained bound on Pt(IV) complex (Figure 6).

The inspiring results of complex **24**, have prompted researchers to fully investigate Pt(IV)-azide complexes as photo-active prodrugs. A series of trans-Pt(IV)-azide complexes with biologically active axial ligands were developed (Shi et al., 2020a). These complexes can selectively target tumor cells depending on the axial ligand and attack multiple cellular components simultaneously, exerting a multi-targeting mechanism of action and thus improving anticancer efficacy. Complexes **25-27a**, replaced one axial hydroxide ligand with dichloroacetate (DCA), coumarin 3-carboxylate (cou), and pyruvate dehydrogenase kinase inhibitor 4-phenylbutyrate (PhB), respectively. They were further functionalized using DCA in place of a second axial hydroxide ligand, obtaining bifunctional complexes **25-27b**, respectively. All these complexes exhibited stronger photocytotoxicity and cell accumulation in A549 cells than the parent complex **24**. Notably, the monofunctional complexes with one axial hydroxide ligand exhibited higher water solubility. At the same concentration, cellular Pt accumulation and light-induced cellular ROS levels were significantly higher for the bifunctional complex compared to the monofunctional complex. Complex **27b** exhibited the highest phototoxicity indices against A549 lung cancer cells. Biotinylated Pt(IV)-azide complexes **28a-b** (Shi et al., 2020b) were also designed to improve therapeutic efficacy *via* biotin mediated lung cancer targeting (Prasad et al., 1999; Wang et al., 1999; Luo et al., 2006). Both complexes were relatively nontoxic to A549 cells and normal MRC-5 lung fibroblasts under dark conditions. Under light irradiation, both complexes exhibited strong photocytotoxicity, and the phototoxicity of the complex **28b** was twice as high as that of complex **28a**.

1,8-Naphthylimine and its extended π-conjugated system are well known as multifunctional fluorescent molecules that have been widely used in oncology treatment (Banerjee et al., 2013; Tandon et al., 2017; Kumari et al., 2019). 1,8-Naphthylimine conjugation with anticancer drugs can enhance the cytotoxicity of the drugs (Chen et al., 2018; Shanmugaraju et al., 2018; Tomczyk and Walczak, 2018; Jia et al., 2019; Ma et al., 2019). Based on this, photoactive Pt(IV) azide complexes **29a-c** with axial 1,8-naphthalimide derivatives were developed (Shi et al., 2021). These complexes were also stable in dark eve in the presence of the bioreductant GSH. Under visible light irradiation, they decomposed to produce significant photocytotoxicity. Complex **29b** bearing electron-withdrawing -NO_2_ substituent exhibited stronger photocytotoxicity than **29c** under blue light irradiation. Notably, complex **29c** bearing electron-donating -NMe_2_ substituent was capable for activation under green light irradiation. Moreover, 1,8-naphthalimide is a photosensitizer that can generate ROS, thus enhancing the photocytotoxicity of photoactive complexes (Banerjee et al., 2013). Exposure of complexes **29a-c** to 465 nm blue light significantly enhanced intracellular ROS level.

**FIGURE 7:**
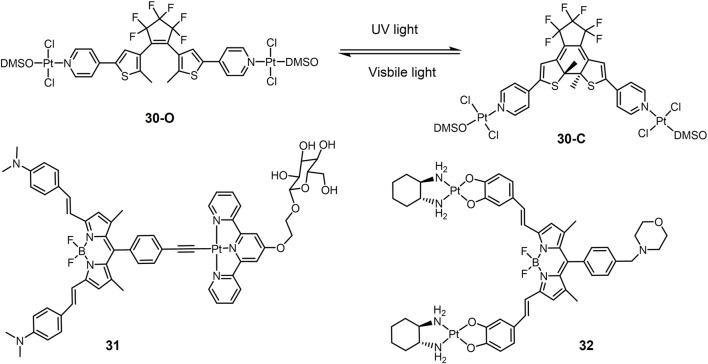
Light-responsive Pt(II) prodrugs bearing chromophores for PACT (**30**) or PDT (**31-32**).

In addition to Pt(IV) complexes, many Pt(II) complexes also exhibit photocytotoxicity. For example, carboplatin and transplatin can dissociate Cl^−^ and convert to substances with higher DNA binding capacity under UV irradiation, which in turn enhances the toxicity to tumor cells (Heringova et al., 2006; Mlcouskova et al., 2012). One of the main strategies for designing photo responsive Pt(II) complexes is the introduction of chromophore moiety harvesting light. Pt(II) complex **30** (Figure 7) contained photoswitchable 1,2-dithienylethene-containing ligands (Presa et al., 2015). 1,2-Dithienylethylene derivatives are photochromic molecules can converted to their open or closed forms upon exposure to visible or ultraviolet light (Irie, 2000). The ring-opening form (**30-O**) and the ring-closed form (**30-C**) exhibited different properties in terms of DNA binding and cytotoxicity. **30-C** interacted more strongly with DNA and modified the DNA shape more efficiently than **30-O**, therefore showing a twice cytotoxicity against small cell lung cancer DMS53 cells (IC_50_ value of 34.4 μM for **30-C** versus 75.9 μM for **30-O**, respectively). These results suggested that complex **30** may be a potential photoactive lung cancer therapeutic agent.

Pt(II) drugs or prodrugs based on cisplatin lack of the ability of generating ROS under photo irradiation. The main strategy to construct Pt(II) drugs for PDT is to combine Pt(II) complexes with photosensitizers. A series of BODIPY-Pt(II) complexes have been developed as potential PDT anticancer agents based on the PDT activity of boron-dipyrrolidene (BODIPY) dyes. The photoactive BODIPY ligand (R-BODIPY) was used to construct Pt(II) complex **31** bearing (Ramu et al., 2018). A glucose group targeting tumor cells was added to the tepyridine molecule in the complex 39 for targeted chemotherapy, in addition to the ability to increase water solubility (Macheda et al., 2005). Under red light (600–720 nm) irradiation, complex **31** showed significant photocytotoxicity against A549 lung cancer cells with negligible dark toxicity.

Although conventional platinum-based anticancer drugs are mononuclear, multinuclear platinum complexes have been reported to show higher anticancer efficacy. A binuclear hybrid ligand Pt(II) complex **32** was developed (Ramu et al., 2019). Complex **32** had a 1,2-diaminocyclohexane ligand and a morpholine-bound BODIPY-linked bridging ligand with two catecholate moieties. A morpholine unit was linked to the BODIPY for lysosomal localization (Andrew et al., 1997; Wang et al., 2016; Zhu et al., 2018). Complex **32** showed low toxicity in the dark and in normal cells and strong photocytotoxicity in A549 lung cancer cells under light (650–850 nm). Cellular localization showed that the complex was mainly enriched in lysosomes. Complex **32** exerted anti-tumor effects by inducing apoptosis through lysosomal dysfunction.

#### 3.1.2 Reactive oxygen species-generating ruthenium complexes

**FIGURE 8:**
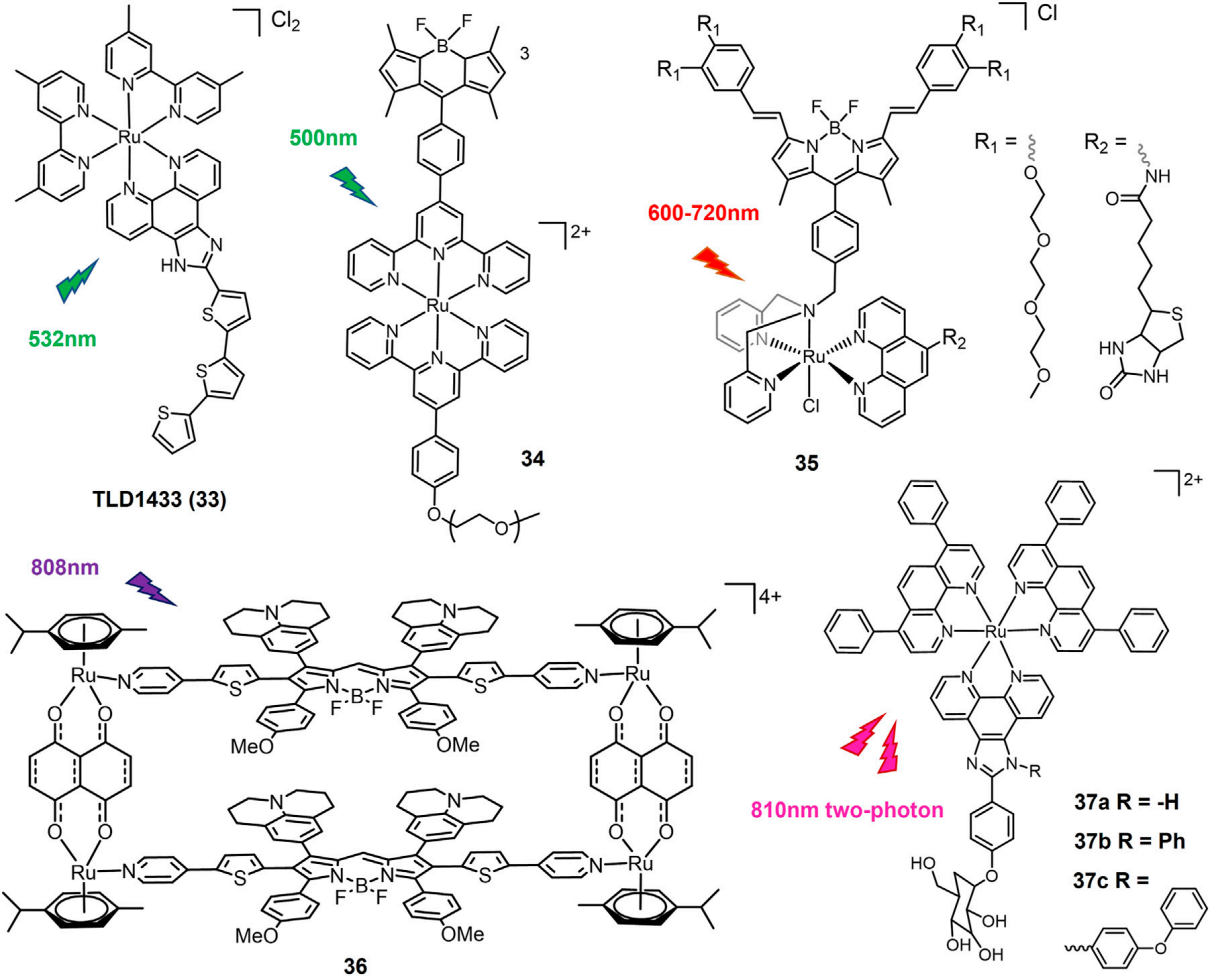
Light-responsive Ru(II) prodrugs as photosensitizers for PDT.

Photoluminescent Ru(II) polypyridyl complexes are considered promising candidates of PSs for PDT due to long-termed photostability and high efficiency of ^1^O_2_ production (Liu et al., 2018). TLD1433 (**33**, Figure 8), the first Ru(II)-based PS that entered a human clinical trial, has successfully completed a phase Ib clinical trials for intravesical PDT of non-muscle invasive bladder tumor (Monro et al., 2019). TLD1433 also shows potentials in combating lung cancer. Under green light (532 nm) irradiation by an optical surface appliactor, TLD1433 induced an EC_50_ of 1.98 μM against A549 cells (Chamberlain et al., 2020). The exciting achievements of TLD1433 encourage researchers to explore luminescent Ru(II) complexes as PSs candidates for lung cancer treatment (Wu et al., 2022).

Notably TLD1433 could not be activated for lung cancer PDT under red light irradiation which has deeper penetration than green light (Chamberlain et al., 2020). To overcome this shortage, long wavelength light activable Ru(II)-based PSs were designed. Complex **34** with BODIPY chromophore conjugated to a Ru(tpy)_2_ complex (Liu et al., 2020a) increased its absorption in the green spectral region (450–600 nm) and promoted the formation of sensitized singlet oxygen upon green light activation, which in turn induced a significant photocytotoxicity against the A549 human lung cancer cell line with an EC_50_ value of 1.50 μM under 500 nm light irradiation at 0.48 J/cm^−2^. Another π-conjugation extended BODIPY-Ru(II) complex **35** was designed and exhibited strong absorption at 604 and 654 nm, lying in the phototherapeutic window (Paul et al., 2020). Complex **35** was activated by low dose of red light (600–720 nm) to show superior phototoxicity with an IC_50_ of 0.02 μM and a PI value of > 5000 against A549 cells. A NIR-II emissive fluorophore was coordinated with dinuclear Ru(II)-arene cocomplex and self-assembled to form metallacycle complex **36** (Xu et al., 2022). With excitation of 808 nm laser, complex **36** emitted over 1000 nm and exhibited photothermal effect. *In vivo* test showed that upon exposure of 808 nm laser, complex **36** efficiently eliminated subcutaneous tumors of A375 cells, showing better therapeutic effect than cisplatin. Recently, this system was employed to design novel Ru(II)-metallacycle that emitted over 1,100 nm, which suggested exciting *in vivo* chemo-phototherapeutic effects for cisplatin-resistant lung cancer (Li et al., 2022).

The Chao group have developed two-photon absorption (TPA) PDT agents based on Ru(II) complexes (Liu et al., 2020b). Complexes **37a-c** exhibited large TPA cross section (σ2) at 810 nm, among which **37c** have the highest σ2 value of 181 GM. Upon 810 nm two-photon irradiation, **37a** showed the strongest phototoxicity against A549 cells and cisplatin-resistant A549R cells, consistent to its highest cellular uptake.

**FIGURE 9:**
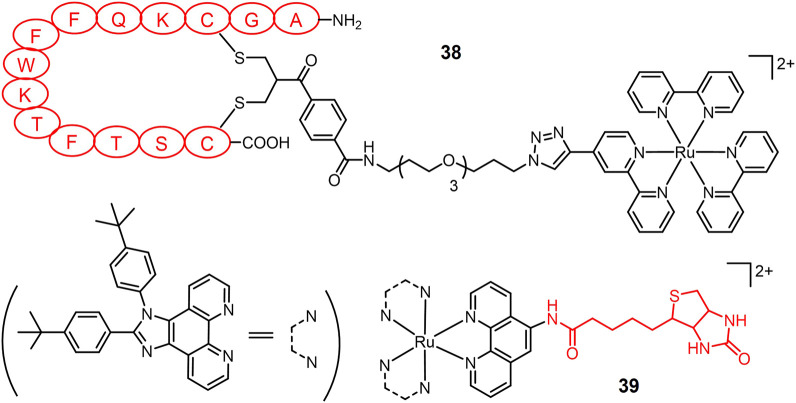
Light-responsive Ru(II) prodrugs bearing receptor-binding ligands as cancer selective photosensitizers for PDT.

Lacking sufficient specificity for cancer cells is one of the major drawbacks of PS for lung cancer PDT. Targeting receptors on lung cancer cells is a useful strategy to design lung cancer selective PSs. Somatostatin receptors (SSTRs) are overexpressed in various cancer cells including lung cancer (Callison et al., 2011). T. Weil and colleagues (Wang et al., 2015b) conjugated a Ru(II)-based PS to the peptide hormone somatostatin (amino sequence: AGCKNFFWKTFTSC, short for SST) *via* an azide-alkyne click reaction to obtain complex **38** (Figure 9). Complex **38** entered cells *via* receptor-mediated endocytosis, exhibiting a 100-fold increased accumulation than non-SST conjugated Ru(II) complex inside A549 cells. Following by light irradiation, complex **38** induced pronounced phototoxicity against A549 cells. The Chao group designed a biotin-modified Ru(II) complex **39** to promote its cancer cell selectivity (Li et al., 2019). Complex **39** exhibited a 13.9-fold increase in intracellular accumulation in A549 cells over normal tissue HLF-a cells. Two-photon irradiation (820 nm) of complex **39** significantly induced apoptosis of A549R cells, with an IC_50_ value of 3.3 μM and a PI value of 22.1.

#### 3.1.3 Photosubstituting ruthenium complexes

Photosensitizers for PDT of lung cancer are relied on oxygen concentration. Making use of photo-induced ligand(s) substitution reaction, ruthenium complex after light irradiation produces an uncaged intermediate that readily coordinates with biomolecules inside cells. These photosubstituting ruthenium complexes serve as PACT agents to combat lung tumors under hypoxia in an oxygen-independent manner.

**FIGURE 10:**
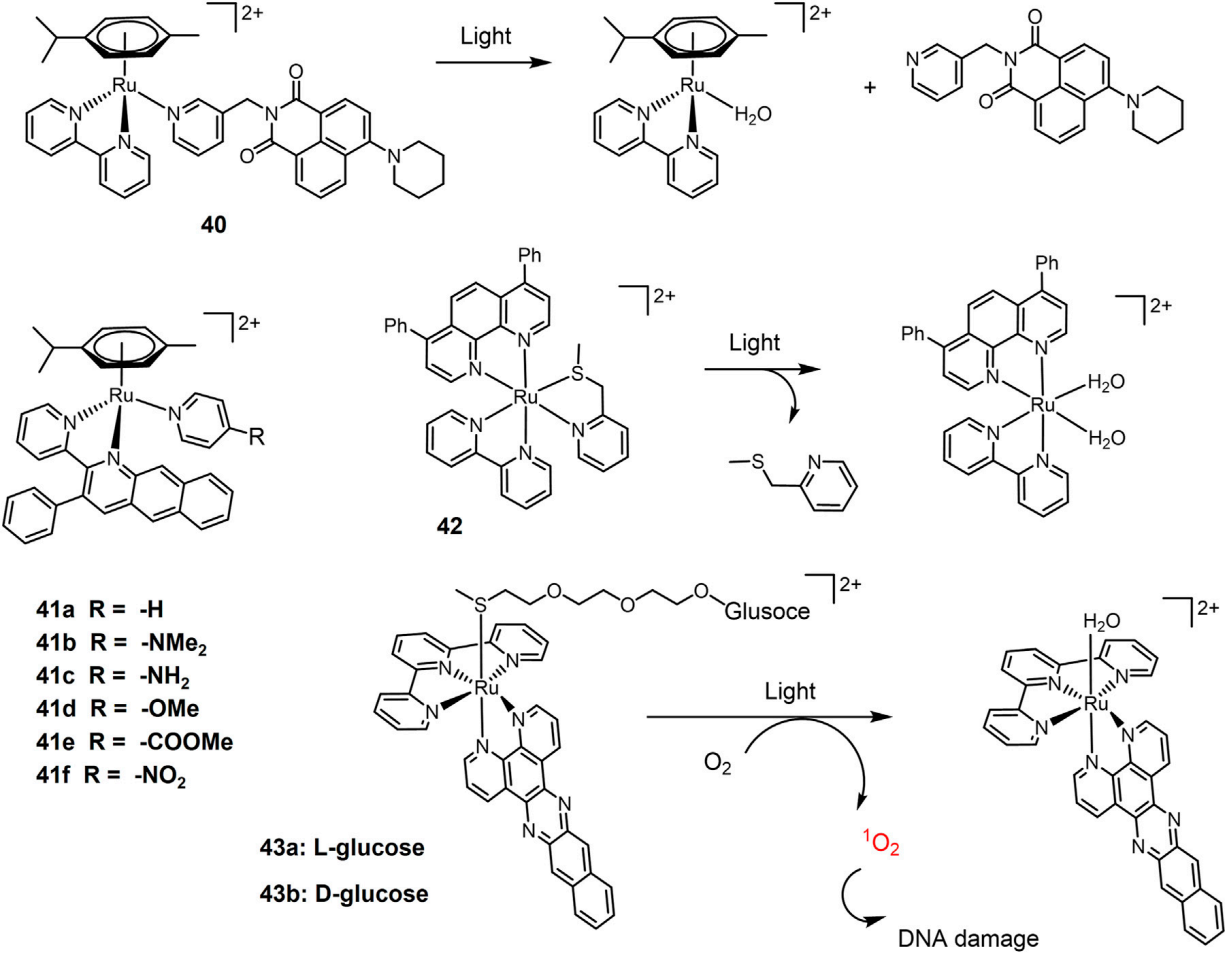
Photosubstituting Ru(II) prodrugs for PACT.

Monodentate ligand in a Ru(II) complex easily undergoes ligand substitution reaction, especially under light irradiation. Half-sandwich Ru(II)-arene complex **40** bearing pyridinyl naphthalimides (Figure 10) as monodentate ligand were stable in the dark, and released the monodentate pyridyl ligand upon blue light irradiation (Bisceglie et al., 2022). It exhibited high photocytotoxicity to A549 cells under blue light irradiation with an IC_50_ value of 10.55 μM. A series of half-sandwich Ru(II) complexes **41a-f** were investigated for synergistic PACT and PDT treatment of lung cancer agent (Chen et al., 2015). Non-substituted complex **41a** exhibited the strongest photocytotoxicity against A549 cells, consistent with its most efficient ability to induce ligand dissociation and ^1^O_2_ production.

The Bonnet group have explored thioether ligand-chelating Ru(II) complexes as PACT agents. The softness of thioether-Ru coordination bonds enhances their photolability (Van Rixel et al., 2016; Cuello-Garibo et al., 2019; Meijer and Bonnet, 2019). A methylthiomethyl pyridine chelating Ru-complex **42** was designed (Chen et al., 2022) and its photocytotoxicity and cellular uptake were evaluated in A549 cells. In the dark, the EC_50_ value was 59 μM. After 15 min of green light irradiation (520 nm, 21 mW/cm-2, 19 J/cm-2), the EC_50_ value (effective concentration) of the complex **42** decreased to 6.5 μM, exhibiting significant photocytotoxicity. By applying such photo-uncaging approach, a rigidin analogue containing thioether group as tubulin polymerization inhibitor, was caged to a Ru-complex (Van Rixel et al., 2019). Upon green light irradiation, rigidin was released, accompanied by drastic reduction of A549 cell growth both *in vitro* under hypoxia and on lung cancer tumor xenograft mice. Caged rigidin on Ru-complexes also reduced its toxicity. Glucose-conjugated complexes **43** were designed to improve their water solubility and cell uptakes (Lameijer et al., 2016). Upon 450 nm light irradiation, the glucose thioester ligand was released and substituted by water, forming a reactive Ru(II) intermediate for DNA interaction. *Via* the introduction of dppn ligand (benzo [i]dipyrido[3,2-a;2′,3′-c]phenazine), both complexes generated massive amounts of ^1^O_2_ for efficient photocleavage of DNA. Dual actions of photo activation mode led to high phototoxicity of **43** under low dose visible light irradiation. Complex **43a** exhibited an EC_50_ value of 0.58 μM against A549 cells with PI value of 86; while **43b** exhibited an EC_50_ (effective concentration) value of 0.72 μM against A549 cells with PI value of 26, respectively.

### 3.2 Thermoresponsive

Another exogenous trigger for metallodrugs is heat. Thermosensitive polymers are often used as carriers because of their solubility that undergoes a structural transition between swelling and contracting types with increasing temperature. Temperature-responsive metallodrugs are constructed by exploiting the temperature difference between diseased and healthy tissues (Neumann et al., 2017). By adjusting the temperature near the low critical solution temperature (LCST) at the tumor location, the polymer can selectively release the metallodrug it carries and thus exert anti-tumor activity (Chen et al., 2016b).

**FIGURE 11:**
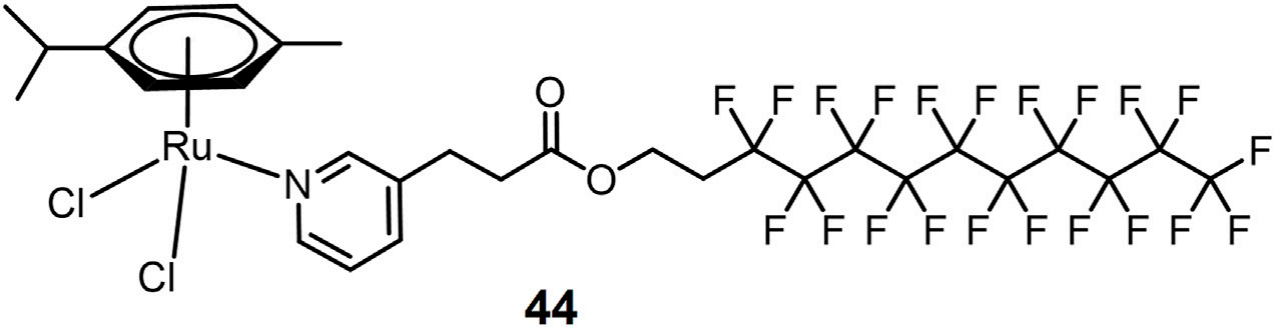
Thermoresponsive Ru(II) prodrug.

A Ru(II) aromatic complex **44** bearing thermoresponsive fluorous chain (Figure 11) has been reported as a heat-sensitive metallodrug for lung cancer treatment (Clavel et al., 2014). A549 cells were tested as a model at 37 and 41°C, respectively. Complex **44** exhibited good thermosensitive behavior. The complex was almost inactive at 37°C closed to normal body temperature with an IC_50_ value > 500 μM. After 2 h of hyperthermia stimulus at 41°C, its toxicity became significant with an IC_50_ value of 33 μM increasing by at least 2 orders of magnitude.

## 4 Dual-stimuli-responsive Pt- and Ru-based anticancer drugs

### 4.1 Redox/pH-responsive

**FIGURE 12:**
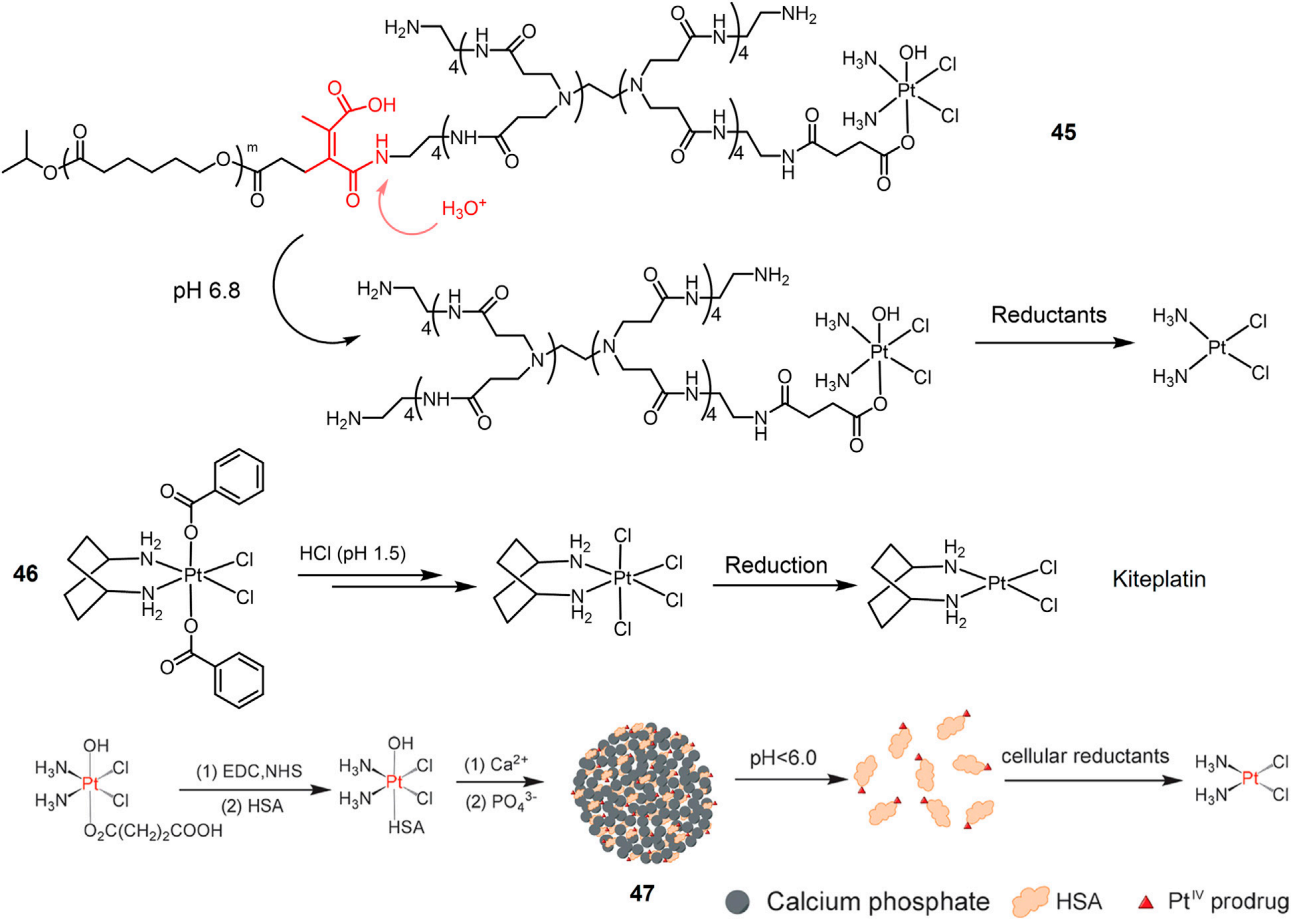
Redox/pH dual-responsive Pt(IV) prodrugs. Schematic illustration of complex **47** was reproduced with permission from ref. (Shi et al., 2015). Copyright 2015 John Wiley.

As mentioned above, Pt(IV) prodrugs are virtually inert until reduced to the corresponding Pt(II) active substance, a property that minimizes adverse side effects. Another approach to improve the efficacy and reduce the side effects of platinum drugs is to load platinum drugs in a drug carrier, thus enabling targeted delivery and release of the drug under specific stimuli. pH-responsive carriers were employed for designing a dual stimuli-responsive metallic prodrugs. A acid-cleavable amide bond was introduced to conjugate a hydrophilic dendrimer loaded with a Pt(IV) prodrug (PAMAM/Pt) with a hydrophobic polymer chain, obtaining **45** (Figure 12) (Li et al., 2016). **45** self-assembled to form clustered nanoparticles with a size of about 100 nm under physiological pH conditions (pH 7.4). Under weak acidic condition (pH 6.8), **45** was hydrolyzed, triggering the release of small PAMAM/Pt prodrugs (∼5 nm). The rapid release of Pt(IV) prodrug with PAMAM dendrimers in acidic tumor microenvironment promoted the tumor penetration of the drug. Finally, Pt(IV) prodrug is rapidly reduced intracellularly to release active cisplatin to produce powerful antitumor efficacy. In animal studies, **45** exhibited extremely effective antitumor effects against A549R cisplatin-resistant human lung cancer model. A dibenzoate-capped kiteplatin analogue (**46**) was stable at acidic condition and substituted by chloride at pH 1.5 mimicking stomach environment. Complex **46** was found significantly more effective than cisplatin against A549 cells *in vitro* with an IC_50_ value of 0.05 μM (Margiotta et al., 2016). *In vivo* anticancer activity of complex **46** after oral administration on LLC-bearing mouse model was comparable with intraperitoneal administration of cisplatin (Barbanente et al., 2022). These results suggest that complex **46** have potential as oral anticancer agents.

pH-responsive nanoparticles were also employed for Pt(IV) prodrugs delivery. A Pt(IV) prodrug was attached to Human serum albumin (HSA) that protected it from premature reduction before being uptaken by cells and improved cancer targeting efficacy (Desai et al., 2006; Neumann et al., 2010; Zheng et al., 2014; Lee et al., 2015). Pt(IV)-HSA was further conjugated with calcium phosphate (CaP) nanoparticles, obtaining a hybrid Pt(IV)-HAS-CaP **47** (Figure 12) (Shi et al., 2015). **47** was decomposed in an acidic environment (pH < 6.0) to release the Pt-HSA complex, which was reduced by reducing agents inside cells to generate active cisplatin, thus exerting anti-tumor effects. Based on the dual response properties, Pt-HSA/CaP is more cytotoxic against A549 cells than cisplatin and the precursor Pt(IV) complex.

In addition to Pt(IV) complexes, Pt(II) complexes can also be used for pH and reduction dual response metal drugs. A dual-responsive supramolecular PEGylated dendrimer system 55 (TSPDS) was reported (Maity et al., 2016). TSPDS has the properties of biocompatibility, monodispersity and highly branched structure (Wolinsky and Grinstaff, 2008; Cheng et al., 2011b; Sun et al., 2015). In addition, TSPDS is formed *via* reducible disulfide polymerization and is redox sensitive (Nystrom and Wooley, 2011). The PEGylated Pt(II) complex (Pt(II)-K-mPEG) is conjugated to TSPDS *via* an acid-dissolvable Pt-O bond (Binauld et al., 2012). The system maintains a stable size of about 120 nm at physiological pH, and at pH 5.0, the Pt-O bond is acidolyzed leading to the release of the Pt(II)-K-mPEG complex. In addition, redox-induced degradation of TSPDS can facilitate the release of acid-activated Pt(II)-K-mPEG complexes under acidic conditions. *In vitro* experiments have shown that lower pH and higher concentrations of reducing agents in A549 cells can trigger the degradation of this system, leading to the release of active platinum analogs for antitumor effects (IC_50_ = 4 μg/ml^−1^). In addition, TSPDS showed comparable antitumor efficacy to cisplatin in A549 xenograft tumors *in vivo* with fewer side effects.

### 4.2 Redox/light-responsive

**FIGURE 13:**
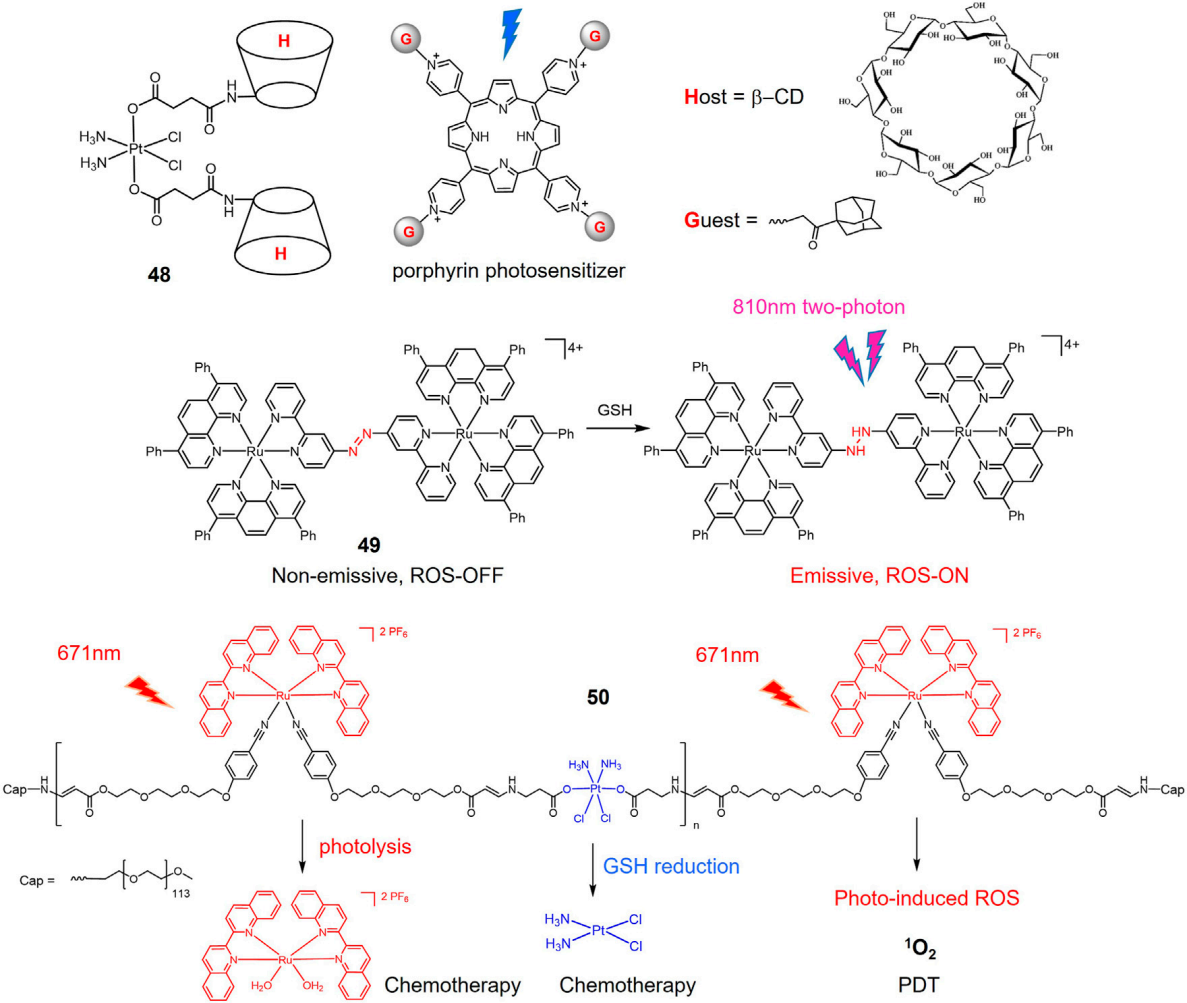
Redox/light dual-responsive Pt(IV) and Ru(II) prodrugs.

The Pt(IV) prodrug-bridged β-CD dimer **48** (Figure 13) is used as the host molecule (H) and the adamantine-modified porphyrin as the guest molecules (G) to construct redox-photo bis-reactive supramolecular self-assembled nanoions (Zhang et al., 2015). The Pt(IV) prodrug is activated by reduction in the presence of reducing agents. The porphyrin-based moieties can act as photosensitizers to generate ROS and exert PDT effects on tumor cells (Detty et al., 2004). Therefore, the supramolecular assembly has potential antitumor activity and overcome cisplatin resistance by dual chemotherapy-photodynamic therapy. The cytotoxicity and cellular uptake of this assembly were evaluated in A549 cisplatin-resistant cells. The results showed that the cytotoxicity was significantly stronger than that of cisplatin under blue light exposure. Moreover, the cellular uptake of the nanoparticles was 12-fold higher than that of cisplatin due to the increased lipophilicity of the porphyrin moiety.

A dinuclear Ru(II)-azo complex **49** was developed as a GSH-activable photosensitizer (Zeng et al., 2017b). The azo-bridged ligand is strongly electron withdrawing and acts as a lock to turn off the luminescence of the complex (Li et al., 2013a; Li et al., 2013b). GSH can reduce azo group in dinuclear Ru(II) complexes into hydrazine group, therefore restoring their luminescence and photo-induced ROS production. *In vitro* experiments showed that the complexes showed high cellular accumulation in the mitochondria of the cells. In A549 cells, the complex showed weak cytotoxicity in the dark (IC_50_ > 70 μM), while its phototoxicity was increased 14-fold (IC_50_ = 5.0 μM) by light irradiation at 450 nm (20 mW/cm^−2^, 15 min).

A Pt(IV) and Ru(II) hybrid polymer **50** containing redox-responsive Pt(IV) moiety and light-responsive Ru(II) moiety was designed as dual-responsive treatment of cisplatin resistant lung cancer (Zeng et al., 2020). Polymer **50** self-assembled into nanoparticles that could be efficiently untaken by cancer cells. Red light irradiation triggered the release of Ru(II) complexes that could bind to DNA for chemotherapy, and generated ^1^O_2_ for PDT. Reduction by GSH inside lung cancer cells activated Pt(IV) prodrug to form cisplatin. Synergistic anticancer actions led to significant phototoxicity against cisplatin-resistant A549R cells.

## 5 Conclusion and perspectives

Stimuli-responsive Pt- and Ru-complexes are promising metallic anticancer candidates to meet the requirements of precision chemotherapy for lung cancer. They allow for targeted drug delivery and activation at the target site, which will help to modulate the efficacy of the drug and avoid side effects. Based on the versatility and ease of modulation, researchers have developed a number of stimulus-responsive Pt- and Ru-complexes for lung cancer using a variety of different stimuli as triggers. In this review, we survey the relevant literature published in the past 20 years and classify the relevant complexes into intrastimulant-responsive, extrastimulant-responsive, and dual-stimulant-responsive metal drugs based on the type of stimulus responsiveness. Typical examples in each category are appropriately presented with respect to their design strategies, mechanisms of reactive action and pharmacological properties.

Despite promising prospects, few stimuli-responsive metallodrugs have entered clinical trials and none has been approved for the treatment of lung cancer. Indeed, there are still a number of issues needed to be addressed in the development of metallodrugs based on Pt-, Ru-complexes, and complexes with other metal ions for lung cancer therapy. One of these challenges is the exploration of new therapeutic targets and their druggability. Currently, most metallodrug researches for lung cancer are focused on targeting DNA. However, this does not always lead to effective drugs because only small number of active drugs successfully address nucleus to exert their DNA binding. Moreover, their exact mechanisms of action and behavior in the biological environment have not been fully explored for many of the metallodrugs on the market. For these reasons, exploring new therapeutic targets and verifying their authenticity is an absolute necessity. Another major challenge is the systemic toxicity and pharmacokinetics of metallodrugs. Although a large number of stimuli-responsive metallodrugs have been found to have different cytotoxic properties *in vitro* and *in vivo*, their pharmacokinetic aspects including absorption, distribution, metabolism and excretion (ADME) properties are less explored. Lipinski’s “Rule of Five” will help to theoretically predict the pharmacokinetics of metallodrugs (Lipinski et al., 2001; Stella et al., 2007). A greater challenge arises from the requirement for precise control and initiation of the response system. For example, the fact that tumor microenvironments only exhibit a tiny difference of pH value compared to the normal tissues requires that the pH response system must be very sensitive to changes in acidity. This demanding requirement also applies to enzyme-responsive metallodrugs. Finally, new mechanisms of action of some non-classical metallodrugs have the potential to be developed into new stimuli-responsive metallodrugs. Regardless of the influence of the ligand on the pharmacological properties of the complex, the metal ion is central to the pharmacological action of the metal complex. However, the vast majority of researches now focuses on transition metal complexes, particularly platinum and ruthenium complexes; while other metal complexes have been largely ignored. The neglected metal complexes may have some unknown properties that could overcome or compensate for the shortcomings of current stimulant-responsive metal drugs. Despite the many difficulties in developing stimulant-responsive metal drugs, this emerging field shows great promise.
